# Cocoa Diet Prevents Antibody Synthesis and Modifies Lymph Node Composition and Functionality in a Rat Oral Sensitization Model

**DOI:** 10.3390/nu8040242

**Published:** 2016-04-23

**Authors:** Mariona Camps-Bossacoma, Mar Abril-Gil, Sandra Saldaña-Ruiz, Àngels Franch, Francisco J. Pérez-Cano, Margarida Castell

**Affiliations:** 1 Department of Physiology, Faculty of Pharmacy, University of Barcelona, 08028 Barcelona, Spain; marionacamps@ub.edu (M.C.-B.); mariadelmar.abril@ub.edu (M.A.-G.); ssaldana@ub.edu (S.S.-R.); angelsfranch@ub.edu (À.F.); franciscoperez@ub.edu (F.J.P.-C.); 2 Nutrition and Food Safety Research Institute (INSA-UB), 08921 Santa Coloma de Gramenet, Spain

**Keywords:** cholera toxin, flavonoids, intestinal sensitization, nutraceutic, oral tolerance, ovalbumin, specific antibodies, Tγδ+ cells

## Abstract

Cocoa powder, a rich source of polyphenols, has shown immunomodulatory properties in both the intestinal and systemic immune compartments of rats. The aim of the current study was to establish the effect of a cocoa diet in a rat oral sensitization model and also to gain insight into the mesenteric lymph nodes (MLN) activities induced by this diet. To achieve this, three-week-old Lewis rats were fed either a standard diet or a diet with 10% cocoa and were orally sensitized with ovalbumin (OVA) and with cholera toxin as a mucosal adjuvant. Specific antibodies were quantified, and lymphocyte composition, gene expression, and cytokine release were established in MLN. The development of anti-OVA antibodies was almost totally prevented in cocoa-fed rats. In addition, this diet increased the proportion of TCRγδ+ and CD103+CD8+ cells and decreased the proportion of CD62L+CD4+ and CD62L+CD8+ cells in MLN, whereas it upregulated the gene expression of OX40L, CD11c, and IL-1β and downregulated the gene expression of IL-17α. In conclusion, the cocoa diet induced tolerance in an oral sensitization model accompanied by changes in MLN that could contribute to this effect, suggesting its potential implication in the prevention of food allergies.

## 1. Introduction

Cocoa powder, derived from *Theobroma cacao* tree seeds, has a mixed composition of over 500 different compounds [1]. It contains macronutrients (carbohydrates, proteins, and lipids, both monounsaturated and saturated fatty acids), fiber (soluble and insoluble), minerals (calcium, cooper, magnesium, potassium), polyphenols (in particular it is rich in flavonoids such as epicatechin, catechin, and procyanidins), and methylxanthines (caffeine and theobromine) [2]*.*

Today, cocoa powder and cocoa products are consumed worldwide [3] and different health benefits have been associated with their consumption [3,4,5]. Cocoa is a rich source of polyphenols, greater than that of tea and wine [3,6], with a potent antioxidant capacity [2,7] due to its phenolic hydroxyl groups [8]. Most of cocoa’s health properties have been attributed to its polyphenol content [3,7] and, in this context, modulation of allergic reactions by several flavonoids has been described [8,9].

Focusing on cocoa and the immune system, previous studies have demonstrated that a 10% cocoa diet has an immunomodulatory effect in the intestinal and systemic immune compartments in rats. Changes in the percentage of B lymphocytes and T cells, including T cell receptor (TCR) αβ+ cells, TCRγδ+ cells, T helper (Th), and T cytotoxic (Tc) cells in mesenteric lymph nodes (MLN), have been described [10,11]. In addition, cocoa diet influences immune functions by modulating cytokine synthesis in MLN cells [12] and attenuating the development of specific IgE, IgG1, IgG2a, IgG2c, and IgM antibodies after intraperitoneal immunization with ovalbumin [12,13]*.*

Food allergies are abnormal immunological reactions to food proteins that generate a wide variety of immune changes and consequently different clinical symptoms and signs [14,15]. The main site of sensitization to food proteins is the gut-associated lymphoid tissue (GALT) [16], which can be classified into inductive sites (Peyer’s patches, isolated lymph nodes, and MLN) and effector sites (lymphocytes in the lamina propria and intestinal epithelium). With regard to unresponsiveness to food antigens, MLN are the primary site for the induction of oral tolerance [17]*.*

Currently, food allergy is becoming a worldwide problem [18]. In particular, its prevalence is increasing in Westernized countries [19]. In this context, oral‑sensitized animal models are of interest in order to assess its mechanisms and to evaluate therapeutic and nutritional interventions. Previously, we set up a model of oral sensitization consisting of the oral co-administration of the food allergen (ovalbumin; OVA) plus cholera toxin (CT) [20]. CT is an effective mucosal adjuvant that breaks down oral tolerance to co-administered protein antigens [21], altering some regulatory mechanisms of the intestinal mucosa [22,23], although the exact pathways involved are still unclear.

Different approaches are used to treat or prevent oral sensitizations [24,25] and, in this sense, nutraceutics could have a potential role. Based on this background, the purpose of the present study was to establish the effect of cocoa consumption, with its recognized immunomodulatory activities, in a rat oral sensitization model. Likewise, in an attempt to gain insight into the mechanisms induced by a cocoa diet, the composition and functionality of cells in MLN were assessed. For these purposes, rats were fed with a 10% cocoa diet for four weeks and for the first three weeks were orally sensitized with OVA and CT. Immune responses were established by specific antibody response during the study as well as by MLN characterization at the end of the study.

## 2. Materials and Methods

### 2.1. Reagents

Albumin from bovine serum (BSA), albumin from chicken egg white (OVA; grade V), CT, gelatine, peroxidase-conjugated extravidin, *o*-phenylenediamine (OPD), 30% hydrogen peroxide (H_2_O_2_), fetal bovine serum (FBS), penicillin-streptomycin, glutamine, Folin-Ciocalteu phenol reagent, gallic acid monohydrate, L-asparagine monohydrate, L-arginine, folic acid, HEPES, and nystatin were purchased from Sigma-Aldrich (Madrid, Spain). Biotin-conjugated anti-rat IgG1, IgG2a, IgG2b, IgG2c, IgM, and IgA monoclonal antibodies were obtained from BD Biosciences (Madrid, Spain). Goat anti-rat IgA, its peroxidase-conjugated antibody, and rat IgA standard were provided by Bethyl Laboratories (Montgomery, TX, USA). Peroxidase-conjugated anti-rat Ig was from Dako Cytomation (Glostrup, Denmark). 2-β-mercaptoethanol, Na_3_N, and paraformaldehyde were purchased from Merck (Darmstadt, Germany). Anti-rat monoclonal antibodies conjugated to a fluorochrome were provided from BD Biosciences (San Diego, CA, USA). Ketamine was obtained from Merial Laboratories S.A. (Barcelona, Spain) and xylazine from Bayer A.G. (Leverkusen, Germany). Dulbecco’s Modified Eagle Medium (DMEM)-GlutaMAX media and gentamicin were obtained from Gibco™ and RNAlater^®^ from Ambion (Thermo Fisher Scientific, Barcelona, Spain). Natural Forastero cocoa was provided by Idilia Foods S.L. (formerly Nutrexpa S.L., Barcelona, Spain) and AIN-93M diet and basal mix by Harlan Teklad (Madison, WI, USA).

### 2.2. Animals and Diets

Thirty-six female Lewis rats were purchased from Janvier Labs (Saint Berthevin, France) and maintained in polycarbonate pathogen-free cages (three rats per cage) with controlled conditions of temperature and humidity and in a 12:12 h light:dark cycle in the Faculty of Pharmacy’s animal facility. All experimental procedures were approved by the Ethical Committee for Animal Experimentation of the University of Barcelona (CEEA/UB ref.5988).

After one week of acclimatization, three-week-old rats were randomly assigned into the following four groups: reference group (RF/R), reference cocoa group (RF/C), sensitized group (OVA/R), and sensitized cocoa group (OVA/C), as detailed in Table 1.

The oral sensitization was performed as previously described [20]. Briefly, rats received orally 50 mg of OVA with 30 µg of CT as adjuvant in 1 mL of distilled water, three times per week (Monday, Wednesday, and Friday) for three weeks. RF/R and RF/C groups received 1 mL of vehicle on the same days. During the 28 days of the study, animals were given free access to water and food. AIN-93M formula was used as the standard diet and a cocoa-enriched diet was produced with the addition of 100 g of defatted cocoa powder to 900 g of a basal mix, the resulting composition finally providing an isoenergetic chow. The two experimental diets provided similar amounts of proteins, lipids, and carbohydrates (Table 2).

### 2.3. Sample Collection and Processing

Blood samples were collected weekly from the beginning of the study. After centrifugation, serum was obtained and frozen at −20 °C until antibody quantification*.*

One week after the last oral administration, rats were anaesthetized with ketamine/xylazine (90 mg/kg/10 mg/kg) and exsanguinated. Urine was collected directly from the urinary bladder with the help of a syringe, and the small intestine and MLN were carefully dissected.

In sterile conditions, MLN were passed through a sterile mesh cell strainer (40 µm, Thermo Fisher Scientific) and the resulting cell suspension was centrifuged (538 g, 5 min, 4 °C) and resuspended with RPMI 1640 medium supplemented with 10% heat-inactivated FBS, 100 IU/mL streptomycin-penicillin, 2 mM l-glutamine, and 0.05 mM 2-β-mercaptoethanol. Cell counting and viability were assessed by Countess™ Automated Cell Counter (Invitrogen™, Thermo Fisher Scientific). Some isolated lymphocytes from MLN were stained to be analyzed by flow cytometry (explained below). Other MLN cells were stimulated *in vitro* to promote cytokine release and the remaining cells were kept in RNAlater^®^ until gene expression analysis. MLN cells were stimulated *in vitro* by culturing 3 × 10^6^ cells/mL in DMEM supplemented with 10% heat-inactivated FBS, 36 mg/L L-asparagine monohydrate, 116 mg/L l-arginine, 10 mg/L folic acid, 500 mg/L HEPES, 10 mg/mL gentamicin, 10,000 U/mL nystatin, 100 U/mL streptomycin-penicillin, and 0.05 mM 2-β-mercaptoethanol. At the same time, a specific stimulus was added (OVA, 10 μg/mL) and, after 72 h, supernatants were collected to assess cytokine production.

The proximal part of the small intestine was opened lengthwise, cut into small pieces, weighed, and incubated in a shaker at 37 °C. After centrifugation, supernatants were collected, aliquoted, and stored at −80 °C until cytokine and IgA quantification.

### 2.4. Determination of Total Polyphenol Content

Total phenolic content was determined according to Folin–Ciocalteu’s method. Briefly, 250 µL of Folin–Ciocalteau’s reagent and 1.25 mL of 20% Na_2_CO_3_ solution were added to 500 µL of diluted urine. After 2 h at room temperature, the absorbance was measured at 765 nm. A standard curve prepared with gallic acid was used.

### 2.5. IgA and Specific Anti-OVA Antibodies

Total serum and intestinal IgA from intestinal lavage were quantified by a sandwich enzyme-linked immunosorbent assay (ELISA), as previously described [26].

Specific anti-OVA antibody (total anti-OVA antibodies and anti-OVA IgG1, IgG2a, IgG2b, IgG2c, IgM, and IgA isotypes) levels were measured by an indirect ELISA. In brief, 96-well polystyrene plates (Nunc Maxisorp^®^, Wiesbaden, Germany) were coated overnight at room temperature with 10 µg/mL of an OVA solution in carbonate buffer (pH 9.6). The plates were washed and blocked with 0.5% gelatin. Afterwards, appropriately diluted samples and standards were added for 3 h. In order to assess total anti-OVA antibodies, peroxidase-conjugated anti-rat Ig and OPD-H_2_O_2_ solution were added. To quantify specific anti-OVA Ig isotypes, biotin-conjugated anti-rat IgG1, IgG2a, IgG2b, IgG2c, IgM, or IgA monoclonal antibodies were used and, thereafter, peroxidase-conjugated extrAvidin and an OPD‑H_2_O_2_ solution were added.

Absorbance was measured in a microplate photometer (LabsystemsMultiskan, Helsinki, Finland) at 492 nm and data was interpolated by Ascent v.2.6 software (Thermo Fisher Scientific). The relative anti-OVA antibody concentration was calculated giving the value of 1 to the mean value obtained from samples from the RF/R group tested in the same conditions and, therefore, all values were expressed as an increase of the mean value of RF/R group.

### 2.6. Immunofluorescence Staining and Flow Cytometry Analysis

Lymphocytes from MLN (5 × 10^5^ cells) were stained using mouse anti-rat monoclonal antibodies conjugated to fluorescein isothiocyanate (FITC), phycoerythrin (PE), peridininchlorophylla protein (PerCP), or allophycocyanin (APC). The antibodies used were anti-CD4, anti-CD8α, anti-CD8β, anti-TCRαβ, anti-TCRγδ, anti-NKR-P1A, anti-CD62L, anti-CD25, and anti-CD103. Cells were incubated with a mixture of saturating concentrations of antibodies in PBS containing 2% FBS and 0.1% Na_3_N, at 4 °C in darkness for 20 min. After washing, cells were fixed with 0.5% p-formaldehyde and stored at 4 °C in darkness until analysis by flow cytometry. A negative control staining using an isotype-matched monoclonal antibody was included in each cell sample. Analyses were performed with a Gallios™ Cytometer (Beckman Coulter, Miami, FL, USA) in the Scientific and Technological Centers of the University of Barcelona (CCiTUB).

### 2.7. Gene Expression from MLN Lymphocytes

Lymphocytes from MLN were kept in RNAlater^®^ until analysis. On the day of RNA extraction, lavages with PBS were performed to remove RNAlater^®^. Immediately, cells were homogenized in a vortex for 2 min. Total RNA was extracted by RNeasy^®^ mini kit (Qiagen, Madrid, Spain) in accordance with the manufacturer’s instructions. RNA quantification was performed with a NanoPhotometer (BioNova Scientific, CA, USA) and reverse-transcribed with TaqMan^®^ Reverse Transcription Reagents (Applied Biosystems, Thermo Fisher Scientific) [27]. Real-time PCR assays (ABI Prism 7900 HT, AB) were performed using specific PCR TaqMan^®^ primers (Applied Biosystems): OX40L (Rn00585582_m1, Inventoried (I)), NF-κB (Rn01399572_m1, I), CD11c (Rn01511082_m1), IL-1β (Rn00580432_m1), IL-12 (Rn00584538_m1), IL-17α (Rn01757168_m1, I), and IL-33 (Rn01759835_m1). The expression of HPRT1 (Rn01527840_m1) was used to normalize the quantification of the studied genes. Relative gene expression levels were calculated using the 2^−ΔΔCt^ method, as previously described [20]. The relative mRNA level was calculated giving the value of 1 to the mean value obtained from samples from the RF/R group tested in the same conditions.

### 2.8. Cytokine Quantification

Interleukin (IL) 4, IL-10, interferon (IFN) γ, and tumor necrosis factor (TNF) α were quantified by BD™Cytometric Bead Assay Rat Soluble Protein Flex Set (BD Biosciences, Madrid, Spain) as detailed in previous studies [13]*.*

### 2.9. Statistical Analysis

Data are expressed as means ± standard error. All statistical analyses were performed with IBM Social Sciences Software Program (SPSS, version 22.0, Chicago, IL, USA).

Levene’s test was performed to assess variance equality, followed by Kolmogorov–Smirnow to determine its distribution. When the results demonstrated equality of variance and normal distribution, a two-way ANOVA test was performed (oral sensitization and diet). When the interaction between oral sensitization and diet was statistically significant, Bonferroni’s *post hoc* test was performed between groups.

Otherwise, when the results had high variance and/or non-normal distribution (food and water intake, anti-OVA antibody concentration, cytokine concentration in MLN cell supernatants), non-parametric tests, such as Kruskal–Wallis and Mann–Whitney U tests were performed. When *p* < 0.05, statistical difference was considered significant.

## 3. Results

### 3.1. Food and Water Intake, Flavonoid Absorption and Body Weight

Food and water intake were monitored throughout the study (Table 3). No differences were found among groups (established by Kruskal–Wallis and Mann–Whitney U tests).

Total polyphenol concentration was quantified in urine samples at the end of the study. Rats fed standard diet showed values ranging between 3.16 and 18.6 µg/mL (mean ± standard error, 10.12 ± 1.63). Cocoa-fed animals had concentrations significantly higher ranging between 26.1 and 61.8 µg/mL (35.86 ± 3.24) (diet effect *p* = 0.000 by two-way ANOVA; no significant effect of oral sensitization, *p* = 0.079, or interaction, *p* = 0.960).

Body weight (Table 3) increased progressively during the study (time effect *p* = 0.000 by two-way ANOVA) and oral sensitization did not affect it (*p* = 0.873 by two-way ANOVA). However, the cocoa diet produced a slower growth (*p* = 0.000 by two-way ANOVA). No significant interactions were found between oral sensitization and diet or time, between diet and time, or between oral sensitization, diet, and time.

### 3.2. Immune Response to OVA: Serum Anti-OVA Antibodies

**Figure 1:**
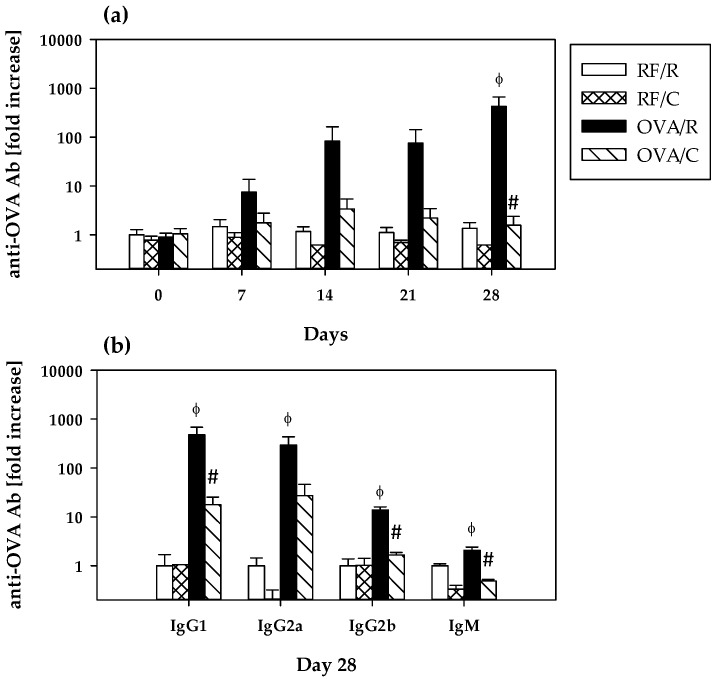
Serum anti-OVA antibodies. (**a**) Total anti-OVA antibody levels throughout the study; (**b**) anti-OVA IgG1, IgG2a, IgG2b, IgM at the end of the study. Values are expressed as mean ± standard error (*n* = 9). Statistical differences: ^ϕ^ *p* < 0.05 compared with RF/R group, and # *p* < 0.05 compared with OVA/R group by Mann–Whitney U test. Groups: RF/R = reference group; RF/C = reference group fed cocoa diet; OVA/R = sensitized group; OVA/C = sensitized group fed cocoa diet.

As shown in Figure 1a, specific total anti-OVA antibodies appeared progressively with the oral sensitization process in the OVA/R group, there being a 7.4-, 82.5-, 75.5-, and 424.5-fold increase with respect to the RF/R group at days 7, 14, 21, and 28, respectively. At the end of the study, total anti-OVA antibodies concentration in the OVA/R group was significantly higher than that in the RF/R group (*p* = 0.038 by Mann–Whitney U test). A total of 78% of animals of the OVA/R group developed antibodies established as mean value of RF/R group plus 2 × SD. On the contrary, levels in the OVA/C group throughout the study were quite similar to those found in the RF/R and RF/C groups (ranging between 1.5- and 3.4-fold increase with respect to the RF/R group) and were significantly lower than those found in the OVA/R group (*p* = 0.035 by Mann–Whitney U test on day 28).

Isotypes of serum anti-OVA antibodies were determined at the end of the study when results could be analyzed with higher sensitivity (Figure 1b). Although no detectable levels of specific IgG2c and IgA were found, the oral sensitization procedure led to the production of anti-OVA IgG1, IgG2a, IgG2b and IgM in such a way that levels were IgG1 (477.8-fold increase of RF/R group) > IgG2a (292.6-fold increase of RF/R group) >> IgG2b (13.9-fold increase of RF/R group) > IgM (2.0-fold increase of RF/R group). These concentrations were significantly higher than those found in the RF/R group (*p* = 0.000, *p* = 0.002, *p* = 0.000, *p* = 0.041 for IgG1, IgG2a, IgG2b, and IgM, respectively, according to Mann–Whitney U test).

In comparison with the OVA/R group, the cocoa-enriched diet significantly attenuated the increase of anti-OVA IgG1, IgG2b, and IgM (*p* = 0.016, *p* = 0.000, *p* = 0.000 by Mann–Whitney U test, respectively) in such a way that concentrations ranged between a 0.5-fold increase for IgM and a 17.9-fold increase for IgG1 of the RF/R group. With regard to IgG2a, although the cocoa diet values were more than 10 times lower than those in the OVA/R group, no statistically significant difference was found with respect to this group (*p* = 0.164 by Mann–Whitney U test).

Anti-OVA antibodies were also analyzed in intestinal lavage but these results were under the limit of detection.

### 3.3. Total IgA Antibodies: Serum and Intestinal Concentrations

**Figure 2:**
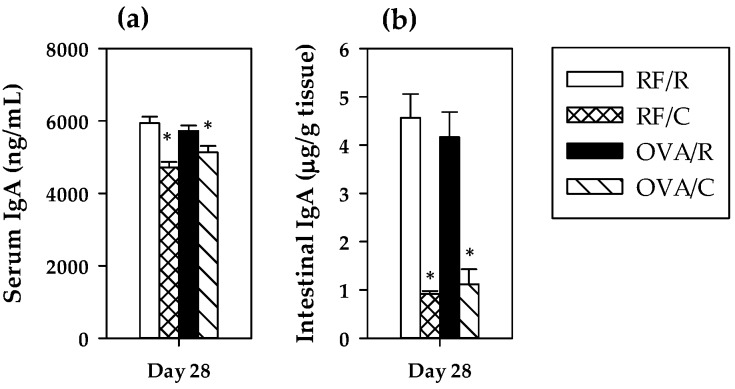
Serum (**a**) and intestinal (**b**) IgA concentrations at the end of the study. Values are expressed as mean ± standard error (*n* = 9). Statistical difference: * means statistical significant difference induced by the diet by two-way ANOVA (*p* < 0.001). Groups: RF/R = reference group; RF/C = reference group fed cocoa diet; OVA/R = sensitized group; OVA/C = sensitized group fed cocoa diet.

To assess the influence of oral sensitization on the main intestinal immunoglobulin, serum and intestinal IgA concentrations were quantified at the end of the study (Figure 2). In both cases, oral sensitization did not significantly modify the IgA concentration (*p* = 0.564 and *p* = 0.830 for serum and intestinal values, respectively, by two-way ANOVA). However, the 10% cocoa diet produced a significant decrease in the IgA levels (*p* = 0.000 in both cases by two-way ANOVA), that was more marked in the intestinal compartment. No significant interaction was found between oral sensitization and diet (*p* = 0.074 and *p* = 0.525 for serum and intestinal values, respectively, by two-way ANOVA).

### 3.4. Lymphocyte Composition of MLN

**Figure 3:**
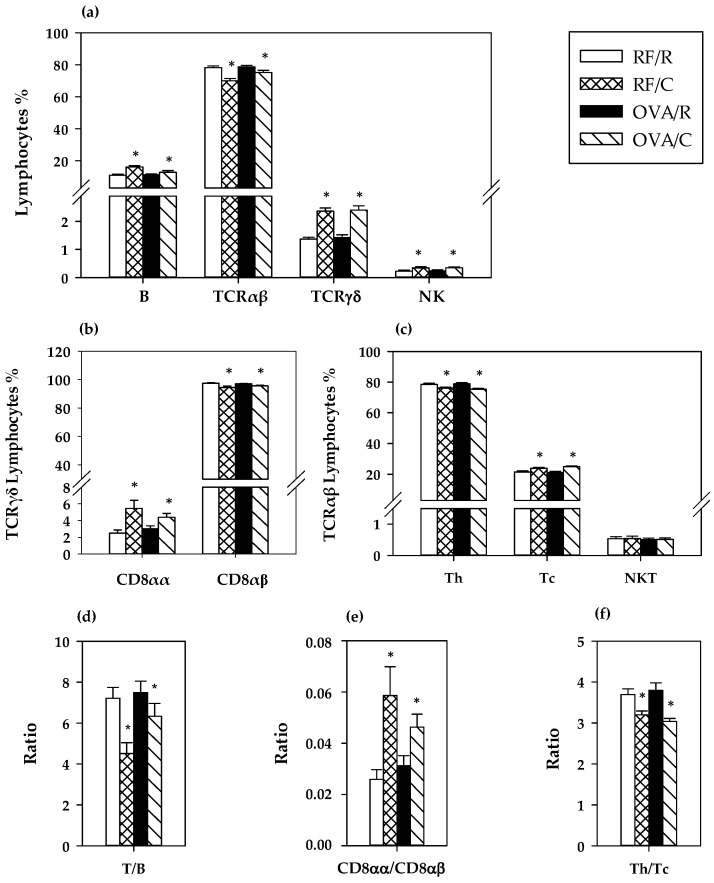
MLN lymphocyte composition. (**a**) Main lymphocyte subsets; (**b**) main TCRγδ+ lymphocyte subsets; (**c**) main TCRαβ+ cell subsets; (**d**) TCRαβ+/B lymphocytes ratio; (**e**) CD8αα/CD8αβ ratio in TCRγδ+ cells; (**f**)Th/Tc ratio in TCRαβ+ cells. Values are expressed as mean ± standard error (*n* = 9). Statistical difference: * means statistical significant difference induced by the diet by two-way ANOVA analysis (*p* < 0.001). Groups: RF/R = reference group; RF/C = reference group fed cocoa diet; OVA/R = sensitized group; OVA/C = sensitized group fed cocoa diet.

The proportion of the main lymphocytes subsets in MLN was established at the end of the study (Figure 3). The oral sensitization did not modify significantly the percentage of B, TCRαβ+, TCRγδ+ and NK cells (*p* = 0.054, *p* = 0.055, *p* = 0.662, and *p* = 0.866, respectively, by two-way ANOVA) in this compartment (Figure 3a). The cocoa diet significantly increased the proportion of B, TCRγδ+, and NK cells (*p* = 0.000, *p* = 0.000, and *p* = 0.007, respectively, by two-way ANOVA) whereas it decreased that of TCRαβ+ cells (*p* = 0.000 by two-way ANOVA) (Figure 3a), producing a lower T/B ratio (*p* = 0.005 by two-way ANOVA) (Figure 3d). The increase of TCRγδ+ cell percentage in animals fed a cocoa diet was due to a higher proportion of CD8αα (*p* = 0.000 according to two-way ANOVA) (Figure 3b,e).

Further analysis of TCRαβ+ cell subsets showed that the reduction in the total TCRαβ+ cell percentage by the cocoa diet was accompanied by an increase in the proportion of Tc cells together with a decrease in that of Th cells (*p* = 0.000 in both cases according to two-way ANOVA) (Figure 3c), which involved a lower Th/Tc ratio (*p* = 0.000 by two-way ANOVA) (Figure 3f). This means that the reduction in TCRαβ+ cell percentage was mainly due to Th cells. No effect on the low percentage of NKT cells was observed by either oral sensitization or cocoa diet (*p* = 0.654 and *p* = 0.930, respectively, by two-way ANOVA).

**Figure 4:**
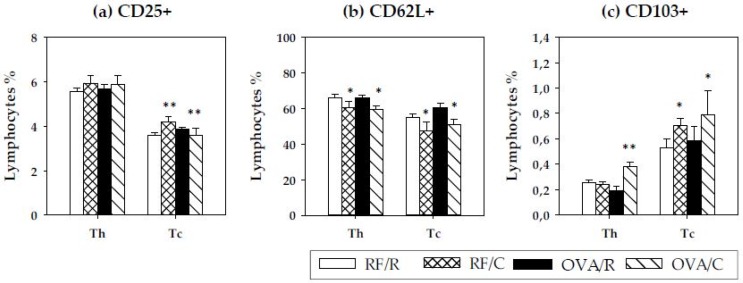
Percentage of cells bearing CD25 (IL2rα), CD62L (L-selectin) and CD103 (integrin αE) in Th and Tc subsets. (**a**) Proportion of CD25+ cells; (**b**) proportion of CD62L+ cells; (**c**) proportion of CD103+ cells. Values are expressed as mean ± standard error (*n* = 9). Statistical differences: * means a statistical significant difference induced by the cocoa diet (*p* < 0.05 by two-way ANOVA); ** means a statistical difference with respect to reference diet (*p* < 0.05 by Bonferroni test). Groups: RF/R = reference group; RF/C = reference group fed cocoa diet; OVA/R = sensitized group; OVA/C = sensitized group fed cocoa diet.

To analyze the Th and Tc subsets in depth, the proportion of activated cells, of cells expressing the l-selectin adhesion molecule and of cells bearing the integrin αE, was determined by means of expression of the clusters of differentiation CD25, CD62L, and CD103, respectively (Figure 4). Regarding activated cells (CD25+ cells), no differences were detected in Th lymphocytes (*p* = 0.912 and *p* = 0.266 by oral sensitization and diet, respectively, according to two-way ANOVA) (Figure 4a). Nevertheless, when considering the percentage of CD25+ cells in Tc lymphocytes, a significant interaction between oral sensitization and cocoa diet was found (*p* = 0.022 by two-way ANOVA), whereas neither condition significantly modified the proportion of CD25+ in Tc cells (*p =* 0.425 and *p* = 0.360 by oral sensitization and diet, respectively, according to two-way ANOVA). Further analysis revealed that CD25+ cell proportion in Tc lymphocytes increased in RF/C animals with respect to the RF/R group (*p =* 0.030 according to the Bonferroni test) but decreased in oral sensitized animals (*p* = 0.031 according to the Bonferroni test).

With regard to the expression of the l-selectin (CD62L+ cells) in Th and Tc cells, a decrease in the percentage of CD62L+ was observed in both subsets as a consequence of the diet (*p* = 0.018 and *p* = 0.013, respectively, by two-way ANOVA) (Figure 4b).

Finally, the proportion of Th and Tc cells bearing the integrin αE (CD103+ cells) was established. In Th cells, there was a significant effect for the interaction between oral sensitization and cocoa (*p* = 0.000 by two-way ANOVA), and the Bonferroni test revealed that there was a higher percentage of CD103+ cells in Th lymphocytes only in oral sensitized animals fed the cocoa diet (*p* = 0.013) (Figure 4c). Considering Tc cells, there was a higher percentage of CD103+ induced by the cocoa diet (*p* = 0.028 by two-way ANOVA).

### 3.5. Gene Expression and Cytokine Production in MLN Cells

The possible influence of the oral sensitization and the cocoa diet on gene expression of some molecules and on cytokine secretion in the MLN lymphocytes was also established.

**Figure 5:**
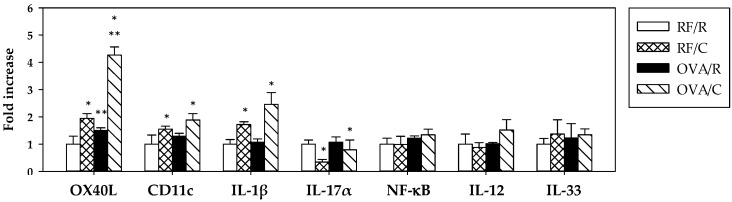
mRNA gene expression in MLN lymphocytes. Values are expressed as mean ± standard error (*n* = 6–9). Statistical differences: * means a significant difference induced by the cocoa diet (*p* < 0.05 by two-way ANOVA), ** means a significant difference with respect to the reference diet or induced by the oral sensitization process (*p* < 0.001 by Bonferroni test). Groups: RF/R = reference group; RF/C = reference group fed cocoa diet; OVA/R = sensitized group; OVA/C = sensitized group fed cocoa diet.

The relative gene expression of molecules associated with dendritic cells (OX40L, CD11c) and representative of an inflammatory response (IL-1β, IL-17α), the regulation of the immune response (NF-κB), the response to antigens (IL-12), and the regulatory function (IL-33) are shown in Figure 5.

Oral sensitization increased the gene expression of OX40L (*p* = 0.000 by two-way ANOVA) and did not modify any of the remaining genes studied. The cocoa diet produced a higher expression of OX40L, CD11c, and IL-1β genes (*p* = 0.000, *p* = 0.018, and *p* = 0.001, respectively, according to two-way ANOVA). There was a significant interaction between oral sensitization and diet for the values of OX40L gene expression (*p* = 0.001 by two-way ANOVA), and further analysis revealed that the expression of this gene was higher in the OVA/C group compared to the OVA/R group (*p* = 0.000 by Bonferroni test).

On the other hand, the cocoa diet decreased IL-17α gene expression (*p =* 0.049 by two-way ANOVA)*.*

To establish the effect of the oral sensitization and the cocoa diet on the cytokine pattern, MLN cells were incubated with OVA (10 µg/mL) for 72 h. From these supernatants IL-4, IL-10, TNF-α, and IFN-γ were quantified (Table 4). Neither oral sensitization nor cocoa intake significantly modified the levels of these cytokines released from MLN cells in the applied conditions, although a tendency to increase IFN-γ and IL-10 was observed in OVA/R animals compared with the RF/R group.

Cytokines were also analyzed in gut lavage. No detectable levels of IL-4, IFN-γ, and TNF-α were found in any group. However, IL-10 was found in the gut lavage of reference animals and it increased because of the oral sensitization process (*p* = 0.000 by two-way ANOVA). There was a significant interaction between oral sensitization and diet (*p* = 0.001 by two-way ANOVA) and further analysis revealed that values of the RF/C group were significantly higher than those in the RF/R group (0.009 by Bonferroni test).

## 4. Discussion

The current study demonstrates that a cocoa diet is able to prevent oral immune sensitization in young Lewis rats. This effect is associated with changes in composition as well as the gene expression of some molecules in MLN that could reflect the induction of tolerance to oral antigens, *i.e.*, the ability to suppress immune reaction to food proteins, through cocoa intake*.*

In the oral sensitization model used, Lewis rats received, by oral route, OVA as allergen and CT as adjuvant to breakdown oral tolerance, as developed previously [20]. For four weeks, rats were fed either a reference diet or a 10% cocoa diet. This amount of cocoa was chosen because previous reports demonstrated the immunomodulatory effect of cocoa at this dose [12,13,28]. The oral sensitization was evidenced by the synthesis of specific antibodies. The antibodies produced in the present study mainly belong to the isotypes related to Th2 responses (IgG1 and IgG2a) [29,30], although a certain amount of anti-OVA IgG2b, related to Th1 responses, was also synthesized. Unlike other animal models that use CT as a mucosal adjuvant with allergens that achieve specific IgE development, such as peanut [31], buckwheat [32], lupin [33], and OVA [34], the model used here does not develop IgE antibodies [20]. The different animal species, the various allergenic molecules, the amount of the adjuvant, or the dosage of immunogen may be responsible for the current lack of specific IgE production. Nevertheless, in the sensitization protocol applied, the cocoa diet was able to attenuate the development of specific IgG1, IgG2b, and IgM, although its effect on anti-OVA IgG2a did not achieve significant differences. These results partially agree with previous studies in food allergy models [13,28] in which cocoa attenuated specific IgG1 and IgG2a antibodies in Brown Norway rats, a rat strain that has shown a different susceptibility to a cocoa diet [35]. In any case, it can be confirmed that a 10% cocoa diet attenuates the production of antibodies and therefore prevents oral sensitization.

Focusing on the intestinal and serum IgA, no differences were seen due to the oral sensitization process, which does not agree with other studies in which CT increases serum and intestinal IgA levels [36]. However, in the current study, a cocoa diet downregulates the production of this immunoglobulin in both compartments and in either reference or sensitization conditions, as in previous studies [11,26,35,37]. Polyphenol content is partially responsible for this effect on intestinal IgA [35]. The attenuation of this immunoglobulin by a cocoa diet seems to be a consequence of a lower homing and activation of IgA+ B cells to the intestinal lamina propria in part due to changes in the gene expression of several molecules [37]. Although intestinal IgA has been associated with oral tolerance [38], our results suggest that it could also be achieved with low levels of this immunoglobulin.

To gain insights into the mechanisms induced by a cocoa diet, we focused on the composition and some functional aspects of MLN cells, due to their important role in oral tolerance [39,40]. The transport of the antigen captured by antigen-presenting cells from lamina propria into MLN is a key point in the induction of oral tolerance [17]. In this compartment, no changes in lymphocyte composition were observed due to the oral sensitization process, which is similar to what was observed in a food allergy model in Brown Norway rats combining an intraperitoneal immunization plus oral administration of OVA [29]. However, the intake of the 10% cocoa diet increased the proportion of B, TCRγδ+, and NK cells, whereas it decreased that of TCRαβ+ lymphocytes, similar to previous studies [11,41].

Interestingly, the cocoa diet induced a higher proportion of TCRγδ+ cells in MLN, which can be attributed to the higher amount of CD8αα+ cells, a typical intestinal phenotype [42], suggesting a possible migration from the intraepithelial compartment to MLN [43]. TCRγδ+ lymphocytes play a crucial role in the mucosal immune system and several studies suggest their function in the induction of tolerance to oral antigens [43,44,45]. In particular, it has been described that the intestinal CD8αα+ TCRγδ+ cells favor tolerance [46], and that the blockade of TCRγδ+ cells results in elevated food allergic responses upon oral sensitization using CT as adjuvant [43]. Moreover, a recent study associates a subset of TCRγδ+ cells with an attenuating effect on the synthesis of antibodies by B lymphocytes [47]. These data could explain why, although we observed a relative increase of B cells in MLN due to the cocoa diet, the levels of specific antibodies in the serum of these animals were low. Therefore, it could be suggested that the increase of TCRγδ+ lymphocytes due to cocoa intake could be partially responsible for the prevention of specific antibody synthesis produced by this diet. In addition, NK cells, which also increased in proportion by cocoa diet, could also contribute to the regulation of antibody synthesis [48].

On the other hand, the cocoa diet decreased the proportion of TCRαβ+ lymphocytes and produced an imbalance in the two main subsets, Th and Tc, in favor of Tc cells, which is also in line with previous studies [11,41,49]. In addition, Th and Tc lymphocytes were characterized according to the surface expression of molecules related to lymphocyte homing (CD62L and CD103) and cell activation (CD25). CD62L, also called l-selectine, is involved in lymphocyte rolling on endothelium and the homing to peripheral lymphoid tissues [50]. The oral sensitization did not modify the expression of this molecule in MLN cells, but the cocoa diet decreased the proportion of both Th and Tc cells bearing CD62L. These results could mean that the cocoa diet decreased the arrival of lymphocytes at MLN and, consequently, their activation, thus avoiding lymphocyte activation and then promoting tolerance. In this context, the study of activated CD25+ cells revealed that cocoa intervention only produced significant changes in the proportion of CD25+ Tc lymphocytes, with opposite effects depending on whether the rats were sensitized or not. Although the proportion of CD25+ cells in Tc increased in healthy conditions, the cocoa diet in oral-sensitized animals decreased the proportion of CD25+ cells in Tc lymphocytes, which could also reflect a decrease in the arrival of these cells, as reflected in the proportion of CD62L+ Tc lymphocytes.

Regarding the molecule CD103, a subunit of the α3β7 integrin that can mediate cell adhesion, migration, and signaling [51], recent studies have demonstrated that it is also important in some resident memory CD8+ cells in various tissues, including the gut [52]. Our data show that the 10% cocoa diet in sensitized animals enhanced the proportion of CD103+ cells, both in Th (CD4+) and Tc (CD8+) cells. Both CD4+103+ and CD8+CD103+ cells have been associated with a regulatory role because their proportion increases after treatment with immunosuppressive agents [53]. Therefore, the increase of these cells in the MLN could contribute to the tolerogenic effect induced by a cocoa diet.

In order to shed some light on the role of MLN in the tolerogenic effects of a cocoa diet in rats, we quantified some genes related to the oral sensitization process, including those of molecules associated with dendritic cells (OX40L, CD11c), and representative of an inflammatory response (IL-1β, IL-17α), the regulation of the immune response (NF-κB), the response to antigens (IL-12), and the regulatory function (IL-33). Firstly, it has been taken into account that MLN gene expression did not produce significant results with regard to the sensitization protocol, with the exception of OX40L. We studied OX40L and CD11c related to dendritic cells because it has been described that there was a selective migration and activation of a unique subset of dendritic cells (CD11c+, CD103+, and CD8+) to the MLN in a model using CT as adjuvant, which seems responsible for Th2 polarization in this model [21]. In spite of these data, our results do not agree with such studies because no increase in cells bearing CD103 and no upregulation of CD11c were found in MLN of orally sensitized rats. In addition, it has been described that CT induces an upregulation of OX40L [21,54] and that OX40L–OX40 interactions led to the generation of Th2 responses during antigen presentation [55,56]. However, other data contradict this role [57]. In the current study, OX40L expression was upregulated by the oral sensitization process, which agrees with data relating this molecule to the induction of a Th2 response [54]. However, surprisingly, the expression of OX40L was also enhanced in rats fed cocoa and had an additive effect on rats administered orally with OVA plus CT. These results would suggest that although OX40L–OX40 interaction is enhanced by oral challenge with a mucosal adjuvant, the role of a cocoa diet could be placed downstream of the Th2 immune responses that would eventually inhibit antibody synthesis. Otherwise, it has been described that the activation of the OX40 pathway can also promote Th1 responses [57], which is in line with the gene expression of IL-1β found to be elevated in cocoa-fed animals. Nevertheless, cocoa has been reported to also possess anti-inflammatory properties [58]*.*

Our results regarding the gene expression of NF-κB do not agree with other studies that report that CT breaks oral tolerance by stimulating the production of NF-κB-dependent proinflammatory cytokines [23]. In agreement with the finding that there is no modification of NF-κB gene expression, we also found that the IL-1β mRNA and the level of TNF-α released by MLN cells from OVA-sensitized animals, typical proinflammatory cytokines, did not change with oral sensitization. The gene expression of IL-12 and IL-33 was also determined because IL-12 is related to the response to antigens and decreases after CT administration [59], and IL-33 is important in the induction of Th2 immune responses [60]. Similar to the above molecules, they were not affected by either oral sensitization or the cocoa diet. Nevertheless, we found that the cocoa diet downregulated the gene expression of IL-17α, which seems to be beneficial to oral tolerance because this cytokine could inhibit the tolerance to antigens [61]*.*

Another aspect studied in the MLN was the release of some cytokines after *in vitro* stimulation. Although we expected to find increased Th2-related cytokines due to the oral sensitization protocol, we found no changes due to either sensitization or the cocoa diet. In this context, Singh *et al.* [62] did not detect changes in the concentrations of IL-4, IL-5, IL-10, and IFN-γ released from OVA-stimulated MLN cells in an oral murine model of food allergy, although a tendency to increase IL-10 and IFN-γ was observed, similar to the results presented here. On the other hand, in gut lavage from the small intestine, we found a rise in IL-10 levels in orally sensitized animals and also after cocoa intake. IL-10 is a multifunctional cytokine that is secreted in Th2 responses [63], in line with our increase in the sensitization protocol, and also plays a role regulating immune response and mucosal tolerance [64], agreeing with the results obtained showing the IL-10 increase in the cocoa-sensitized group.

Finally, although further research is necessary to establish the cocoa component responsible for its effects on the immune system and the prevention of oral sensitization, the possible role of flavonoids must be considered, particularly the flavanols, which are abundant in cocoa. In this context, the preventive effects of several flavonoid compounds and classes in allergy have been described [8] and, more recently, it has been reported that epicatechin and also a cocoa extract rich in epicatechin are able to decrease allergic symptoms, including the attenuation of specific antibodies, in a model of orally sensitized mice with OVA together with CT [62]. Therefore, even though more studies are necessary, the epicatechin present in cocoa appears to be one of the cocoa compounds able to prevent oral sensitization in rats. On the other hand, further studies must also be carried out in allergic humans in order to extrapolate the tolerogenic effect of cocoa on this process.

## 5. Conclusions

In conclusion, the cocoa diet, due to its flavanol content such as epicatechin or other compounds, is able to induce tolerance in an oral sensitization model in rats. Changes in mesenteric lymph node lymphocytes, particularly a higher proportion of TCRγδ+ (CD8αα+) and CD103+CD8+ cells and a lower proportion of CD62L+CD4+ and CD62L+CD8+ cells, together with the regulation of some immune-related genes, could contribute to this effect. These results show the ability of a cocoa diet to prevent the breakdown of oral tolerance and its potential as a nutraceutic in food allergies.

## Figures and Tables

**Table 1:** Experimental design.

Group	Oral Administration Days 0, 2, 4, 7, 9, 11, 14, 16, 18, and 21	Diet
RF/R (*n* = 9)	Vehicle (1 mL/rat)	AIN-93M
RF/C (*n* = 9)	Vehicle (1 mL/rat)	10% cocoa
OVA/R (*n* = 9)	50 mg OVA + 30 µg CT/rat	AIN-93M
OVA/C (*n* = 9)	50 mg OVA + 30 µg CT/rat	10% cocoa

**Table 2:** Composition of the diets.

Components	Reference Diet	10% Cocoa Diet	
	AIN-93M (g/kg Diet)	Basal Mix (g/kg Diet)	Cocoa Powder (g/kg Diet)
Carbohydrates	721.9	692.5	16.8
Proteins	140.8	118.2	23.1
Lipids	38.7	27	11.5
Fiber	50	24.5	35.6
Micronutrients	48.6	37.8	6.3
Flavonoids ^1^	0	0	4.02
Theobromine	0	0	2.5
Total	1000	1000	

^1^ total polyphenol content was determined according to the Folin–Ciocalteu method. The cocoa used in this study contained 40.18 mg/g of total polyphenols (expressed as catechin). Reversed-phase high performance liquid chromatography coupled to a diode array detector revealed that cocoa contained 2.34 mg/g epicatechin and 0.4 mg/g catechin.

**Table 3:** Body weight (g) and food and water intake (g/100 g rat/day) from the four groups over the study. Data represent mean ± standard error (*n* = 9 for body weight, *n* = 3 for water and food intake established in each cage). Statistical difference: * means statistical significant difference induced by the diet by two-way ANOVA (*p* < 0.001).

Day	Variable	Group ^1^			
		RF/R	RF/C	OVA/R	OVA/C
0	Body weight	59.9 ± 4.27	59.78 ± 4.58	60.86 ± 4.24	58.62 ± 4.16
0–7	Food intake	10.38 ± 2.35	13.46 ± 1.12	9.72 ± 2.50	13.43 ± 0.82
0–7	Water intake	12.06 ± 0.17	23.85 ± 4.03	12.06 ± 1.81	23.04 ± 3.04
7	Body weight	92.7 ± 6.05	82.28 ± 5.30 *	94.00 ± 5.88	80.90 ± 4.80 *
7–14	Food intake	11.65 ± 0.97	13.05 ± 0.51	11.72 ± 1.04	12.77 ± 0.33
7–14	Water intake	9.68 ± 0.90	21.08 ± 3.11	10.68 ± 0.65	22.64 ± 2.10
14	Body weight	129.04 ± 5.96	107.82 ± 5.84 *	129.03 ± 6.06	109.35 ± 4.53 *
14–21	Food intake	9.63 ± 1.03	11.12 ± 0.95	9.07 ± 0.92	11.24 ± 0.12
14–21	Water intake	9.12 ± 0.26	17.26 ± 0.09	9.13 ± 0.38	21.39 ± 0.99
21	Body weight	154.29 ± 3.72	133.80 ± 5.24 *	152.73 ± 5.67	135.55 ± 4.63 *
21–28	Food intake	6.79 ± 1.86	9.74 ± 0.51	6.82 ± 1.99	8.8 ± 1.36
21–28	Water intake	9.11 ± 0.10	14.99 ± 0.53	10.24 ± 0.96	25.45 ± 4.04
28	Body weight	174.13 ± 3.23	153.32 ± 5.67 *	171.61 ± 4.35	150.53 ± 2.57 *

^1^ Groups: RF/R (reference group: vehicle and AIN-93M diet); RF/C (reference cocoa group: vehicle and 10% cocoa diet); OVA/R (sensitized group: OVA plus CT and AIN-93M diet); and OVA/C (sensitized cocoa group: OVA plus CT and 10% cocoa diet).

**Table 4:** Cytokine production from OVA-stimulated MLN lymphocytes and from gut lavage. Values are expressed as mean ± standard error (*n* = 6–9). Statistical difference: ** means a statistical difference with respect to reference diet (*p* < 0.01 by two-way ANOVA); # *p* < 0.05 compared with RF/R group by Bonferroni test. N.D. means non detectable levels.

Sample	Cytokine	Group ^1^			
		RF/R	RF/C	OVA/R	OVA/C
MLN	IFN-γ	1.000 ± 0.110	1.484 ± 0.661	2.158 ± 0.843	1.521 ± 0.323
MLN	IL-4	1.000 ± 0.080	0.807 ± 0.113	0.895 ± 0.083	0.768 ± 0.110
MLN	TNF-α	1.000 ± 0.065	0.860 ± 0.040	1.033 ± 0.087	0.901 ± 0.089
MLN	IL-10	1.000 ± 0.042	1.106 ± 0.133	1.584 ± 0.302	1.241 ± 0.033
gut lavage	IFN-γ	N.D.	N.D.	N.D.	N.D.
gut lavage	IL-4	N.D.	N.D.	N.D.	N.D.
gut lavage	TNF-α	N.D.	N.D.	N.D.	N.D.
gut lavage	IL-10	1.000 ± 0.586	17.512 ± 3.783 ^#^	25.177 ± 3.151 **	17.060 ± 3.739

^1^ Groups: RF/R (reference group: vehicle and AIN-93M diet); RF/C (reference cocoa group: vehicle and 10% cocoa diet); OVA/R (sensitized group: OVA plus CT and AIN-93M diet); and OVA/C (sensitized cocoa group: OVA plus CT and 10% cocoa diet).

## Abbreviations

The following abbreviations are used in this manuscript:

APC	allophycocyanin
BSA	albumin from bovine serum
CT	cholera toxin
DMEM	Dulbecco’s Modified Eagle Medium
ELISA	enzyme-linked immunosorbent assay
FBS	fetal bovine serum
FITC	fluorescein isothiocyanate
GALT	gut-associated lymphoid tissue
IFN	interferon
I	inventoried
Ig	immunoglobulin
IL	interleukin
MLN	mesenteric lymph nodes
NF	nuclear factor
OPD	*o*-phenylenediamine
OVA	ovalbumin
OVA/C	sensitized group fed cocoa diet
OVA/R	sensitized group
PBS	phosphate-buffered saline
PCR	polymerase chain reaction
PE	phycoerythrin
PerCP	peridininchlorophylla protein
RF/C	reference group fed cocoa diet
RF/R	reference group
Tc	T cytotoxic
TCR	T cell receptor
Th	T helper cells
TNF	tumor necrosis factor
